# Reinforcement of the Standard Therapy with Two Infusions of Convalescent Plasma for Patients with COVID-19: A Randomized Clinical Trial

**DOI:** 10.3390/jcm11113039

**Published:** 2022-05-27

**Authors:** Joan Bargay-Lleonart, Fiorella Sarubbo, Maria Arrizabalaga, José Maria Guerra, Josep Borràs, Khaoulah El Haji, Magdalena Flexas, Jorge Perales, Victoria Fernández-Baca, Carmen Gallegos, Manuel Raya Cruz, Sonia Velasco, Víctor López, Ana Cruz, Antonia Bautista-Gili, Teresa Jimenez-Marco, Enric Girona-Llobera, Laia Vilaplana, Laura Calonge, Juan Tena, Maria Pilar Galán, Antoni Payeras

**Affiliations:** 1 Hematology Department, Son Llàtzer University Hospital, Crta. Manacor Km 4, 07198 Palma, Spain; jbargay@hsll.es (J.B.-L.); jmguerra@hsll.es (J.M.G.); jbartborras@hotmail.com (J.B.); m.flexas@ssib.es (M.F.); jorge.perales@hsll.es (J.P.); svelasco@hsll.es (S.V.); vlopez1@hsll.es (V.L.); 2 Faculty of Medicine, University of the Balearic Islands, 07122 Palma, Spain; 3 Study Group of Monoclonal Gammapathies and Myelodysplastic Syndromes, Health Research Institute of the Balearic Islands (IdISBa), Crta. Manacor Km 4, 07198 Palma, Spain; 4 Health Research Institute of the Balearic Islands (IdISBa), 07198 Palma, Spain; khaoulah.elhaji@ssib.es; 5 Research Unit, Son Llàtzer University Hospital, Crta. Manacor Km 4, 07198 Palma, Spain; 6 Faculty of Science, University of the Balearic Islands, 07122 Palma, Spain; 7 Internal Medicine Department, Son Llàtzer University Hospital, Crta. Manacor Km 4, 07198 Palma, Spain; marrizab@gmail.com (M.A.); manuelraya@hotmail.com (M.R.C.); acruz@hsll.es (A.C.); apayeras@hsll.es (A.P.); 8 Microbiology Department, Son Llàtzer University Hospital, Crta. Manacor Km 4, 07198 Palma, Spain; vfernandez-baca@hsll.es (V.F.-B.); mcgallegos@hsll.es (C.G.); 9 Multidisciplinary Sepsis Group, Health Research Institute of the Balearic Islands (IdISBa), 07198 Palma, Spain; 10 Study Group of Infectious Diseases-VHI, Health Research Institute of the Balearic Islands (IdISBa), 07198 Palma, Spain; 11 Blood and Tissue Bank Foundation of the Balearic Islands (FBSTIB), Cr. Rosselló i Cazador, 20, 07004 Palma, Spain; abautista@fbstib.org (A.B.-G.); tjimenez@fbstib.org (T.J.-M.); egirona@fbstib.org (E.G.-L.); 12 Public Health Research Group of the Balearic Islands (GISPIB), Health Research Institute of the Balearic Islands (IdISBa), Crta. Manacor Km 4, 07198 Palma, Spain; 13 Internal Medicine Department, Hospital Comarcal Manacor, Crta. Manacor Alcudia, 07500 Manacor, Spain; lvilaplana@hmanacor.org; 14 Internal Medicine Department, Mateu Orfila Hospital, Menorca, Rda. de Malbúger, 1, 07703 Mahón, Spain; laura.calonge@hgmo.es; 15 Hematology Department, Mateu Orfila Hospital, Menorca, Rda. de Malbúger, 1, 07703 Mahón, Spain; juan.tenaanies@hgmo.es (J.T.); pilar.galan@hgmo.es (M.P.G.)

**Keywords:** COVID-19, hyperimmune plasma, convalescent plasma, donors, antibody, randomized clinical trial

## Abstract

Background: The aim was to evaluate the reinforcement of the standard therapy with hyperimmune plasma (HP) in Coronavirus-19 disease (COVID-19) patients. Methods: Open-label, multicenter, randomized clinical trial performed in three hospitals in the Balearic Islands. Non-severe COVID-19 hospitalized patients with clinical time evolution equal to/less than 7 days were included, and randomized in: plasma group (PG) (*n* = 37), receiving 600 mL divided into two doses from convalescent plasma donor, administered on days 1 and 2 after the enrollment; and control group (CG) (*n* = 17). Primary outcome was the time for clinical improvement within 21 days, defined as patient achievement of categories 8, 7, and 6 in the Adaptive COVID-19 Treatment Trial scale (ACTT). The trial was terminated early due to the impossibility of recruitment due to the pandemic. Results: PG presented better scores on the ACTT scale at 7 days after HP infusion, whereas CG was needed 14 days to achieve similar results. The plasma infusion was safe. Conclusions: Despite the tendency observed in the plasma group to achieve slightly earlier better physical condition compared with the standard treatment alone. The administration of HP has been shown to be a safe therapy. No robust evidence was found to affirm a therapeutic effect of the early administration of two infusions of HP for non-severe COVID-19 infected patients. The interpretation is limited by the early termination of the trial, which resulted in a small sample size.

## 1. Introduction

The Coronavirus Disease 2019 (COVID-19), whose etiological agent is the Severe Acute Respiratory Syndrome Coronavirus 2 (SARS-CoV-2), is a respiratory illness that emerged in December 2019 in China. It was rapidly spread throughout the world, generating a “Pandemic of Alarming Levels of Spread and Severity” according to the World Health Organization (WHO) [1]. The virus shares similar pathogenesis and symptomatology with pneumonia induced by the Severe Acute Respiratory Syndrome Cove (SARS-CoV) or the Middle East Respiratory Syndrome Cove (MERS-CoV), belonging to the *Coronaviridae* family [2]. The symptomatology of COVID-19 ranges from asymptomatic carriers to severe disease characterized by sepsis and acute respiratory failure. The most frequent symptoms include shortness of breath, dry cough, and fever. Diagnosis is made by the detection of SARS-CoV-2 via reverse-transcriptase-polymerase-chain-reaction (RT-PCR) [3]. Currently, there is not an effective antiviral treatment for COVID-19, although a great number of drugs and treatments have been evaluated since the beginning of the pandemic [4], among them antiviral agents, inflammation inhibitors/antirheumatic drugs, low molecular weight heparins, hyperimmune immunoglobulins, and the hyperimmune plasma (HP) from convalescent patients [5,6,7,8].

HP therapy, which is a classic adaptive immunotherapy, has been applied for the prevention and treatment of many infectious diseases for more than one century, having a satisfactory efficacy and safety in the treatment of SARS, MERS, and the 2009 H1N1 pandemic [9,10]. At the beginning of the pandemic and until the onset of the vaccine, it was hypothesized about the possible efficacy of HP therapy, since SARS-CoV-2 share virological and clinical similarities with SARS and MERS, adding to their relative ease of obtaining, low cost, and security [11]; therefore, patients who had recovered from COVID-19, with a high neutralizing antibody titer, were seen as a valuable source of HP [7]. A great number of clinical trials using HP in different patient types and a dosage were initiated, opening the debate on its effectiveness [12] and the consequent demand for the scientific community to explore this strategy [7,11]; however, most of the studies did not reach the desired sample size because of the uncertain nature of the pandemic, including the rollout of the vaccines, which affected the methodological framework. Researchers, aware of the situation, agreed on the need to quickly share the results achieved so far. Hence, were share the results of Coplasma-2020, a controlled randomized clinical trial, whose objective was to assess the efficacy of the reinforcement of the standard treatment for COVID-19 with two doses of HP from convalescent COVID-19 HP donors, transfused to hospitalized non-severe COVID-19 patients.

## 2. Materials and Methods

### 2.1. Trial Design

We conducted a multicenter randomized controlled clinical trial from July 2020 up to September 2021, when the last patient completed follow-up, at the following hospitals of the Balearic Islands: Son Llàtzer University Hospital (HUSLL) and Regional Hospital of Manacor (RHM) in Majorca, Mateu Orfila Hospital (MOH) in Menorca, with the collaboration of the Blood & Tissue Bank Foundation of the Balearic Islands (FBSTIB). The trial was approved by the Ethical Committee of Balearic Islands (CEI-IB) (code IB4207/20PI). 

### 2.2. Recruitment and Selection Criteria

**Figure 1:**
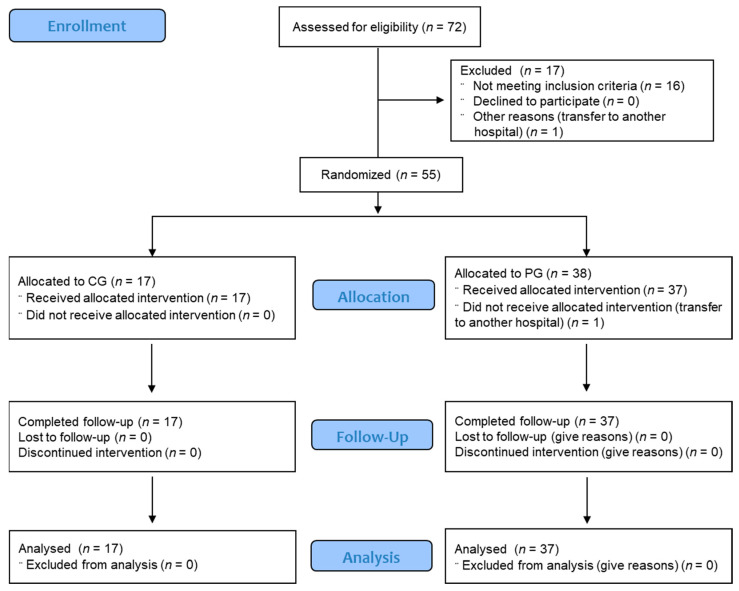
Patient Screening, Enrollment, Randomization, Follow-up, and Analysis Population.

For patient recruitment, the following eligibility criteria were established: (1) patients with age greater than or equal to 18 years, male or female, hospitalized in a COVID-19 area (non-intensive care where COVID-19 patients were hospitalized for specific management and isolation from other patients), with a positive diagnosis by RT-PCR assay for SARS-CoV-2 on nasopharyngeal swabs at the time of the screening; (2) presence of respiratory symptoms and/or fever associated with COVID-19, with clinical time evolution equal to or less than 7 days (day 1 being the day of symptom onset, and including day 7), presence of pneumonia on chest X-ray and/or SatO2 < 94% aa, and Sequential Organ Failure Assessment score (SOFA) < 6; (3) ability to understand the patient information sheet and sign written informed consent, accepting the condition of complying with the procedures established in the protocol. The exclusion criteria were: (1) patients with a previous history of allergic reaction to any blood component; (2) pregnant or lactating women; (3) patients treated with plasma within 21 days prior to the screening; (4) patients with incompatible conditions, e.g., being a participant in other clinical trials. The patient withdrawal criteria were: (1) incomplete study procedures; (2) study written informed consent withdrawal; (3) lack of data collection; (4) deceased during the follow-up period. Patients were informed that they may withdraw from the study at any time without affecting their future medical care. For information on recruitment, see the consort recruitment flow chart (Figure 1).

### 2.3. Randomization and Intervention

Eligible patients were randomly assigned to one of the following groups: plasma group (PG) and control group (CG). The plasma group (PG) received 600 mL, divided into two doses of 300 mL of HP, from a convalescent plasma donor with ABO group compatibility, administered by peripheral or central intravenous route every 24 h on days 1 and 2 from the treatment group assignment. The 600 mL of plasma came from a single donor patient following the transfusion principle of minimizing the risk of adverse reactions to transfusions by assuring a low exogen antigen exposition. Each plasma bag (300 mL) contained an IgG greater than 50 UA/mL against SARS-CoV-2 spike (S) protein (determined by a chemiluminescence assay for the quantitative determination of specific anti-S1 and anti-S2 IgG antibodies against SARS-CoV-2, Liaison SARS-CoV-2 S1/S2 IgG). The infusion of each plasma bag had a duration of 1.5 to 2.0 h. The control group (CG) did not receive plasma intervention. Both groups received the standard therapy according to medical indication, following the local clinical guidelines for the management of COVID-19 patients established in the recruitment period. The standard therapy consists of the use of the following drugs: antivirals, antibiotics, glucocorticoids, anticoagulants, and oxygen therapy. According to the national guidelines for the management of COVID-19 at the time of recruitment [13], without statistical differences in administration or dosage in CG and PG. For specific information about the standard treatment, see Supplementary Data Table S1. For the concomitant pathologies’ treatment, the administration of the usual medication was allowed. A random number computer system (Microsoft Excel^®^ specific matrix for randomization) generated a randomization sequence with a balanced permuted block design (block size 2). Randomization was performed in a proportion of 2:1 (plasma group: control group).

#### Intervention: HP and Plasma Donors

HP was obtained from plasma donors who, after having recovered from the disease, had a negative diagnosis by RT-PCR assay for SARS-CoV-2 on nasopharyngeal swabs, and that fulfilled the compliance with the criteria to be accepted as a plasma donor in accordance with the European Directive (EU) 2016/1214 and the Spanish regulations (RD 1088/2005) for transfusions. Donors were identified through the hospital’s register. The eligibility criteria for donors were: male with age equal to or greater than 18 years with the ability to understand the patient information sheet and sign written informed consent of the study, and presence of detectable anti-SARS-CoV-2 IgG antibodies in peripheral blood greater than 50 AU/mL, with an absence of symptoms due to COVID-19 in the last 14 days, and negative RT-PCR SARS-CoV-2 in at least one nasopharyngeal sample. Weight over 50 kg and good venous access. The exclusion criteria for donors were: have had plasmapheresis in the last 7 days, blood donation in the last 7 days, and donation of more than 25 L of plasma in the previous 12 months. The mean ± SEM time to negativization in plasma donors was 22 ± 2.2 days (time elapsed from a positive PCR up to the first negative PCR in one infection episode), and the mean ± SEM time elapsed from negativization up to donation was 75 ± 7.6 days, with an IgG titer range of 50–195 AU/mL. With the intention of reducing the risk of transfusion-related acute lung injury, only male donors who have never been transfused were selected. Potential donors who provided written informed consent were examined by a hematologist to verify compliance with the inclusion criteria. In order to facilitate the donation process, two large donation groups participated in the study, with the same eligibility and exclusion criteria. One was selected and recruited at the HUSLL, being the donations of this group used in plasma receptors hospitalized in the same hospital. The other group was selected and recruited at the FBSTIB, which provides HP to the RHM and MOH.

### 2.4. Clinical and Laboratory Monitoring

The patients’ clinical status was monitored daily until their discharge from the hospital by trial physicians specialized in internal medicine. The monitoring time points were set at 3, 7, 14, and 21 days after the screening and inclusion in the study. Information was recorded into an electronic database. Supplementary Materials Table S2 synthesizes the procedures. Epidemiological and baseline parameters were: registered age, sex, body index mass (BMI), concomitant illnesses and medication, smoke/abuse drugs habits, and the standard treatment for COVID-19. Vital signs, including diastolic/systolic blood pressure, temperature, cardiac and respiratory frequency, and oxygen saturation, were also recorded. Venous blood samples were collected for biochemistry, hematological determinations, coagulation, IL-6, and IgG titer determination. Serum samples were preserved at −20 °C until the completion of the trial. The levels of anti-S IgG SARS-CoV-2 were analyzed using a chemiluminescence assay for the quantitative determination of specific anti-S1 and anti-S2 IgG antibodies against SARS-CoV-2 (Liaison SARS-CoV-2 S1/S2 IgG). COVID-19 diagnosis was assessed by RT-PCR assay for the qualitative detection of nucleic acid from SARS-CoV-2 on nasopharyngeal swabs (TaqPath COVID-19 RT-PCR, Thermo Fisher, Waltham, MA, USA). Hospital-stay days and the days to achieve negativization were also recorded. Patients were also monitored for adverse events.

### 2.5. Trial End Points

It was difficult to establish an endpoint as the primary outcome in the course of a situation as complex as a pandemic; therefore, the Adaptive COVID-19 Treatment Trial scale (ACTT) version II proposed by the Regulatory Agencies of the National Institute of Allergy and Infectious Disease of the United States and the Remdesivir trial from China [14], was used as primary endpoint [15]. This is an ordinal scale of eight categories of COVID-19 severity based on the patient’s physical situation, whose design was based on a blueprint of the WHO in treating COVID-19 [16]. The primary endpoint of ACTT is the time to recover or achieve clinical improvement, where recovery is defined as the first day on which the subject satisfies one of the following three categories from the ordinal scale: (1) Not hospitalized, no limitations on activities (Point = 8); (2) not hospitalized, limitation on activities, and/or requiring home oxygen (Point = 7); (3) hospitalized, not requiring supplemental oxygen—no longer requires ongoing medical care (Point = 6) [14]. The other categories of the scale are: (4) hospitalized, not requiring supplemental oxygen—requiring ongoing medical care (COVID-19 related or otherwise) (Point = 5); (5) hospitalized, requiring supplemental oxygen (Point = 4); (6) hospitalized, on non-invasive ventilation or high flow oxygen devices (Point = 3); (7) hospitalized, on invasive mechanical ventilation or extracorporeal membrane oxygenation (ECMO) (Point = 2); (8) death (Point = 1). For more information on the scores featured in this scale, see Table S3 in the Supplementary Data and the Primary Outcome Measures of the clinical trials with the Identifiers: NCT04492475 and NCT04280705 at ClinicalTrials.gov (retrieved the 24 May 2022).

### 2.6. Early Trial Termination

The trial was initiated with an elevated number of cases of COVID-19 in the Balearic Islands. Then, with the onset of vaccines, patients’ symptomatology changed, and the evolution of the pandemics fluctuated; therefore, it became clear that it would take approximately several months to reach the enrollment goal. Consequently, with an enrollment of almost 30% of the target population, we decided that it would be logistically impossible and ethically questionable, based on the trial complexity and cost, to continue the trial, and so we ended the trial in order to examine the results.

### 2.7. Statistical Analysis

The trial was designed in April 2020 and initiated in July 2020 due to the need to achieve ethical approval of the protocol. At this time, there was very limited information for sample size estimation. We calculated that by accepting an alpha risk of 0.05 and a beta risk of 0.2 in a two-sided test, 63 subjects were necessary in the CG and 126 in the PG to find a statistically significant proportion difference, expected to be 0.6 in group 1 and 0.8 in group 2, with an anticipated drop-out rate of 3%. The arcsin approximation was used. Following the model of other clinical trials [17], the estimation was calculated considering an upgrade of 20% in the achievement of the three better categories of the ACTT scale, which was the primary outcome at 7 days after enrollment. The analysis was performed with the Granmo^®^ software, Version 7.12 April 2012, IMIM, Barcelona, Spain. For the analyses of registered data, descriptive analyses of variables were performed. Continuous variables are presented as means and standard deviations of the mean (SEM) or medians and interquartile ranges. Categorical variables are presented by frequencies and percentages. For the relationship between variables, the following tests were performed: χ^2^-test and Fisher test for the comparison between categorical variables; after assessing normality, two-way ANOVA repeated measures followed by the post hoc Tukey test when compared the evolution over time of continuous variables in each group were conducted; a one-way ANOVA followed by the Tukey post hoc test for the comparison of the mean of day 21 vs. baseline in continuous parameters was also performed. For correlation between variables, the Pearson test was used. For statistical analyses, Graphpad v.8 software, Version 7.12 April 2012, IMIM, Barcelona, Spain, was used.

## 3. Results

### 3.1. Epidemiological and Baseline Characteristics of the Trial Population

A total of 54 patients that met the eligibility criteria, including patients having tested positive for SARS-CoV-2 infection and that signed the informed consent, were included in the study and underwent randomization. In total, 37 were assigned to the plasma group (PG) and 17 were assigned to the control group (CG). There were no clinically significant imbalances in baseline characteristics between the PG and CG. The mean ± SEM of age was 58 ± 2 years for PG and 59 ± 3 for CG, being men 63% of patients in the PG and 59% in the CG. BMI was 30.72 ± 1.24 for PG and 30.34 ± 1.99 for CG. Concomitant illness was presented in 80% of CG and 73% of PG. The concomitant medication for these pathologies was 45.9% in PG and 41.2% in CG. No toxic habits, including smoking and drug abuse, were present in 94% in PG and 94% in CG. As a standard treatment for COVID-19, following the clinical guidelines, the following types of medications were used in each group: anti-inflammatory, 34.1% in PG and 32.3% in CG; antivirals, 16.5% in PG and 14.7% in CG; antibiotics, 11.8% in PG and 8.8% in CG; anticoagulants, 15.3% in PG and 14.7% in CG; oxygen therapy 22.35% in PG and 29,4% in CG (Table 1).

### 3.2. Primary EndPoint

**Figure 2:**
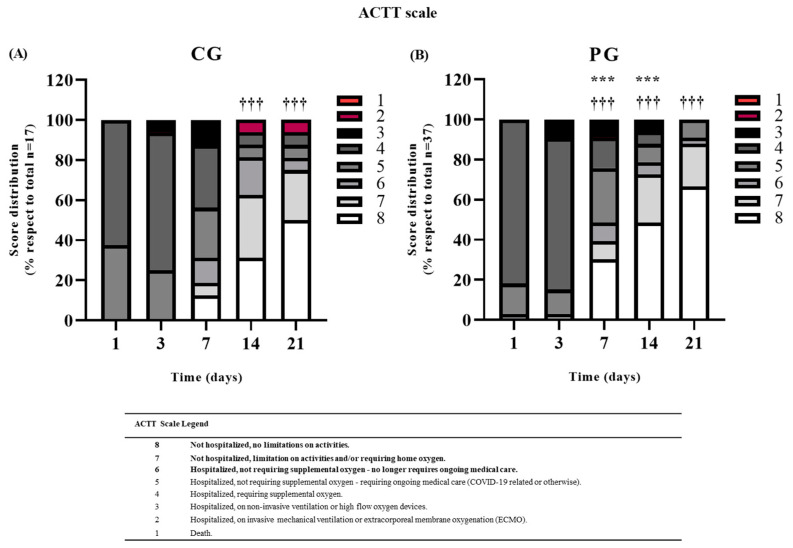
Distribution of the Adaptive COVID-19 Treatment Trial scale (ACTT) score results in: (**A**) control group (CG) *n* = 17 and (**B**) plasma group (PG) *n* = 37, over the follow-up period (1, 3, 7, 14, and 21 days after the first infusion). Bars represent the percentage of patients with respect to the total in each group that present each score (from 8 to 1) in the time-point mentioned. The meaning of each score is explained in the ACTT Scale legend. *** *p* < 0.001, when using the χ^2^ test to compare the scores at 7, 14, and 21 days with respect to day 1 in each group; ^†††^ *p* < 0.001, when using the χ^2^ test to compare the scores at 7 and 14 days in the PG with respect to the scores at 7 and 14 days in the CG. Scores considered as a clinical improvement are marked in bold.

As shown in Figure 2 the ACTT Scale is categorized into eight scores; the meaning of each score is explained in the ACTT scale legend and in Table S3 in the Supplementary Materials. A positive evolution in a patient was recognized when the patient presented categories 8, 7, or 6 of the ACTT scale. For comparisons, we considered the time needed to achieve one of these categories. We observed a positive evolution in both groups, but the PG was considered significant from day 7 with respect to their baseline measurement (χ^2^= 19.45, df = 1, *p*-value < 0.001), being maintained over time at 14 days (χ^2^ = 37.26, df = 1, *p*-value < 0.001) and 21 days (χ^2^ = 57.74, df = 1, *p*-value < 0.001). In contrast, in the control group, the first significant evolution with respect to their baseline was observed from day 14 (χ^2^ = 14.12, df = 1, *p*-value < 0.001), and also maintained at 21 days (χ^2^ = 15.57; *p*-value < 0.001). Comparing PG vs. CG, we observed a significant difference between groups at 7 (χ^2^ = 11.24; df = 1; *p*-value < 0.001) and 14 days (χ^2^ = 23.14; df = 6; *p*-value < 0.001), with better results in the scale for the PG.

### 3.3. Immunity

**Figure 3:**
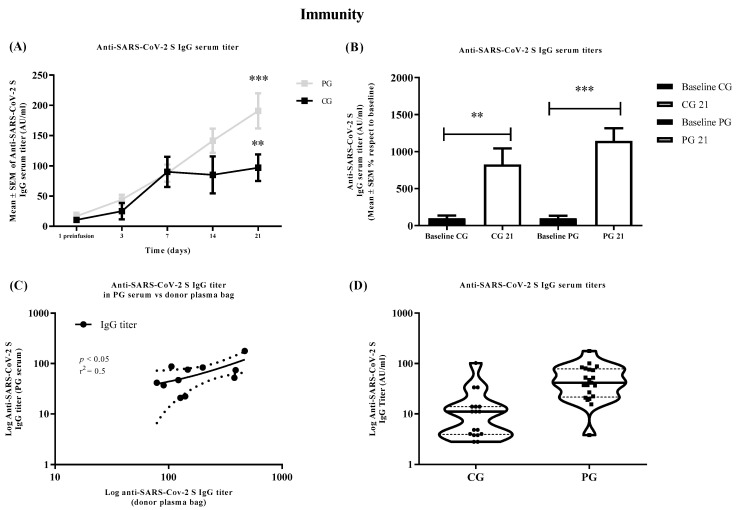
Changes in immunity parameters at baseline, 1, 3, 7, 14, and 21 days after the first plasma infusion comparing the control group (CG) with the plasma group (PG). (**A**) Evolution of anti-SARS-CoV-2 S IgG serum titer (AU/mL) in CG and PG patients, expressed as mean ± SEM at each time point. *** *p* < 0.001, ** *p* < 0.01, when compared by two-way ANOVA repeat measures followed by the post hoc Tukey test, the levels of anti-SARS-CoV-2 S IgG serum titer at 21 days with respect to the baseline in each group. (**B**) Comparison of the anti-SARS-CoV-2 IgG serum titer (AU/mL) at 21 days respect the baseline, expressed as mean ± SEM, in CG and PG. *** *p* < 0.001, ** *p* < 0.01, when compared by one-way ANOVA followed by the post hoc Tukey test, the levels of anti-SARS-CoV-2 S IgG serum titer at 21 days with respect to the baseline in each group. (**C**) Correlation between the levels of anti-SARS-CoV-2 S IgG titers in PG serum vs. donor plasma bag received at 24 h after the last infusion; the correlation was performed using the Pearson test r^2^ = 0.5, *p* < 0.05. (**D**) Distribution of the levels of anti-SARS-CoV-2 S IgG titers (AU/mL) in each group 24 h after the last plasma infusion. The horizontal bars indicate medians and the dotted line interquartile ranges. Each circle represents one patient.

As can be seen in Figure 3A, after enrollment and over the follow-up, there was an increase in the levels of anti-SARS-CoV-2 S IgG serum, which was significant for both groups (F(4, 142) = 14.63, PG *p* < 0.001 and CG *p* < 0.001), according to two-way ANOVA repeat measures followed by the post hoc Tukey test. These results are corroborated when comparing only the levels of anti-SARS-CoV-2 S IgG titer at 21 days vs. baseline in PG and CG, and is also significant for both groups (F(3, 54) = 24.87, PG *p* < 0.001 and CG *p* < 0.001), according to one-way ANOVA repeat measures followed by the post hoc Tukey test (Figure 3B). Although low, due to the number of participants, the correlation tendency between the anti-SARS-CoV-2 IgG titer in PG serum 24 h after the last infusion and the donor plasma units was positive, assessed by Pearson (r^2^ = 0.5, *p* < 0.05) (Figure 3C). Finally, Figure 3D shows the distribution of the levels of anti-SARS-CoV-2 IgG in CG and PG.

### 3.4. Follow-Up Clinical Parameters

**Figure 4:**
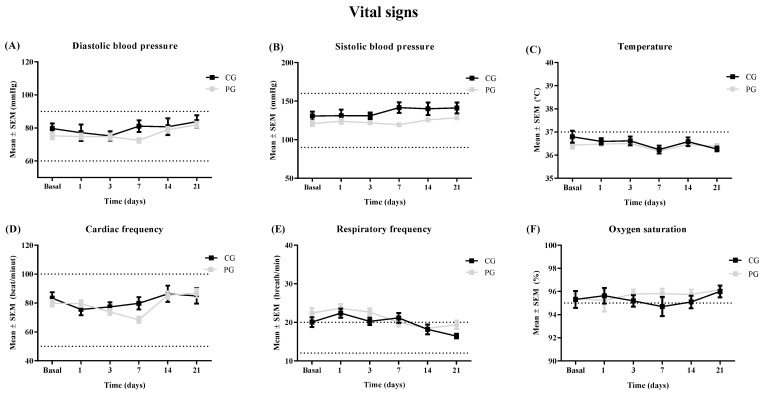
Evolution of the vital signs in control group (CG) and plasma group (PG), from baseline and at 1, 3, 7, 14, and 21 days after the enrollment, including: (**A**) diastolic blood pressure (mmHg), (**B**) systolic blood pressure (mmHg), (**C**) temperature (°C), (**D**) cardiac frequency (beat/min), (**E**) respiratory frequency (breath/min), and (**F**) oxygen saturation (%). Each variable is expressed as the mean ± SEM obtained in each group. There were no statistical differences by one-way ANOVA followed by the Tukey post hoc test.

The time of hospitalization was similar in both groups, being 8.9 ± 0.9 days for PG and 10.5 ± 1.8 for CG. Regarding the time required to obtain a negative PCR, there were no differences between groups, with 12.4 ± 1.4 days in PG and 14.6 ± 2.1 in CG. The administration of HP was secured, and there were no detected significant adverse events (AAEE) due to the plasma infusion. The few AAEE registered were skin rash (grade 1) resolved in 1 day, hearing loss in the left ear, mild dizziness, and left hypochondrium pain, all of them in 1 patient of 37 (2.7%) (Table 2). Figure 4 shows the evolution of diastolic and systolic blood pressure, temperature, cardiac and respiratory frequency, and oxygen saturation, registered before the plasma infusion and during the follow-up time periods (3, 7, 14, and 21 days after infusion) in both groups. There were no significant differences between groups or over time in all parameters (Figure 4F). Hematimetry, coagulation, and biochemical variables were also assessed, but no statistical differences were found over time or between groups (see Supplementary Figures S1–S3). During the enrollment and follow-up, no patients were derived to an intensive medical care unit or death.

## 4. Discussion

Although the present results glimpsed that the reinforcement of the COVID-19 standard therapy with two doses of HP in non-severe infected patients may have a tendency to reduce the probability of progression to a more severe presentation of the disease one week after the infusion of HP, the result’s interpretation should be considered with caution due to the early termination of the trial that caused a small sample size. Coinciding with other studies, HP was a safe therapy [11,18,19,20], and no differences were found in other endpoints [19,21,22]. Our results are in line with the information summarized by Jorda et al. [23]—sixteen clinical trials focused on the use of plasma for the treatment of COVID-19. The clinical improvement was analyzed in four out of the sixteen trials, varying the definition of clinical improvement among them and without consensus on the results; therefore, the hypothesis of an effect on the time of clinical improvement was downgraded due to concerns regarding the risk of bias or incomplete sample recruitment, as is the case with this trial. Furthermore, among the set of trials analyzed in patients with COVID-19, treatment with convalescent plasma, when compared with control, was not associated with lower all-cause mortality or improved disease progression in vital signs. 

This trial has several differences compared to others focused on HP therapy for COVID-19 [11,12,18,21]. At the beginning of the pandemic, when this trial was designed, there were high rates of mortality. Consequently, studies were predominantly focused on the effect of HP in severe patients [11,24]. They concluded without consensus, since some authors affirmed that plasma therapy did not result in significant improvement [11,24], but others associated HP with mortality reduction [25,26] and faster clinical recovery [20]. It should also be considered that many patients suffered from severe conditions after being hospitalized as moderate infected patients; these patients were the most common patients admitted to hospitals. Some trials have suggested that the benefit of plasma therapy is associated with its application in the early stages of COVID-19 [7,18,27,28]. Accordingly, prevention in the progression to severe stages of the disease was a target; therefore, we focused on patients with a top time infection evolution of 7 days at the enrollment’s time with moderate severity, testing the effect of two infusions of HP. There is also a lack of consensus since the literature has suggested a dose-dependent IgG effect of HP, based on the idea that high-titer plasma therapy could reduce the risk of severe respiratory disease, and antibodies could be the main active therapeutic component in convalescent plasma [18,29]. Although, it was also stated that high-titer convalescent plasma did not improve survival or other prespecified clinical outcomes. 

Most cited studies suggested that the results should be considered with caution, pending final consensus, because they were carried out in an unconventional situation, in the context of continuously unpredictable situations, which altogether made the execution of any study difficult due to the lack of knowledge about the behavior of the study population, changes in the hospitals’ organization, the pandemic containment policies, and the epidemiological behavior of the pathogen. On top of those factors, the onset of vaccination programs drastically reduced the percentage of infected patients and their severity. These explain the lack of statistical power to ascertain long-term outcomes in published trials [11,18,21]. Nonetheless, the authors of these studies agreed to publish their results in order to underscore the need to define the effect of classical IgG therapies as helping delay infection progression. In this sense, our study contributes to the aim of assessing the utility of immunological therapies, and the implementation of these trials will boost plasmapheresis programs, improving the self-management capacity of blood components by encouraging the need for speedy donations and management of the same. 

Currently, the use of HP is authorized only in the framework of clinical or observational trials [12]. As the present results are merely descriptive, many questions remain unanswered about the molecular mechanisms of the intervention; therefore, as other studies demonstrated [30], a deeper approach is needed for assessing not only the efficacy of HP in different conditions but also for understanding the action of humoral immunity against of SARS-CoV-2 virus, in comparison with other kinds of endogenous and exogenous immunological strategies.

## 5. Conclusions

Despite the tendency observed in the plasma group to achieve slightly earlier better physical condition compared with the standard treatment alone, and that the administration of HP has been shown to be a safe therapy, no robust evidence was found to affirm a therapeutic effect of the early administration of two infusions of HP for non-severe COVID-19 infected patients. The interpretation is limited by the early termination of the trial, which caused a small sample size; however, results are shared with the scientific community, as is the case with all similar clinical trials. More studies are needed to reach a consensus on the use of plasma therapy in COVID-19.

## Figures and Tables

**Table 1:** Epidemiological and baseline characteristics of the trial population, randomized in plasma group (PG) and control group (CG).

Epidemiological and Baseline Parameters	PG (*n* = 37)	CG (*n* = 17)	Epidemiological and Baseline Parameters	PG (*n* = 37)	CG (*n* = 17)
**Age** (years)	58 ± 2	59 ± 3	**Concomitant medication**		
**Sex**			Yes	17/37 (45.9%)	7/17 (41.2%)
Women	14/37 (37%)	7/17 (41%)	No	20/37 (54.1%)	10/37 (58.8%)
Men	23/37 (63%)	10/17 (59%)	**Smoke**		
**BMI**	30.72 ± 1.24	30.34 ± 1.99	Yes	2/37 (6%)	1/17 (6%)
			No	30/37 (94%)	16/17 (94%)
			**Abuse drugs**		
**Concomitant ilnesses**			Yes	2/37 (6%)	1/17 (6%)
Yes	27/37 (73%)	14/17 (80%)	No	30/37 (94%)	16/17 (94%)
No	10/37 (27%)	3/17 (20%)	**Standard treatment**		
**Type of disease**			Antiinflammatorory	29/37 (34.1%)	11/17 (32.3%)
Cardiac diseases	32 (86.5)	16 (94.1)	Antiviral	14/37 (16.5%)	5/17 (14.7%)
Respiratory diseases	10 (27)	6 (35.3)	Antiobiotic	10/37 (11.8%)	3/17 (8.8%)
Digestive diseases	3 (8.1)	3 (17.6)	Anticoagulant	13/37 (15.3%)	5/17 (14.7%)
Neurological diseases	8 (21.6)	5 (29.4)	Oxygen theraphy	19/37 (22.3%)	10/17 (29.4%)
Psychiatric diseases	0 (0)	5 (29.4)			
Nephrological diseases	8 (21.6)	3 (17.6)			
Oftalmological diseases	1 (2.7)	2 (11.8)			
Dermatological diseases	0 (0)	1 (5.9)			
Endocrinological diseases	36 (97.3)	15 (88.2)			
Rheumatic/osteoarticular diseases	5 (13.5)	5 (29.4)			
Inmunitary diseases	0 (0)	1 (5.9)			

**Table 2:** Clinical parameters and AAEE in plasma group (PG) and control group (CG).

Clinical Parameters	PG (*n* = 37)	CG (*n* = 17)
Stay in hospital (days)	8.9 ± 0.88	10.5 ± 1.84
Time to negativization (days to achieve negative PCR)	12.4 ± 2.14	14.6 ± 1.40
**AAEE(n/N total) %**	4/37 (10.8%)	
**Type of AAEE**		
Skin rash (Grade 1)	1/37 (2.7%)	
Left ear hearing los	1/37 (2.7%)	
Mild dizziness	1/37 (2.7%)	
Left hypochondrium pain	1/37 (2.7%)	

## Data Availability

Data supporting reported results can be found at ClinicalTrials.gov Identifier: NCT04803370 (accessed on 24 May 2022).

## Supplementary Materials

The following supporting information can be downloaded at: https://www.mdpi.com/article/10.3390/jcm11113039/s1, Figure S1. Changes in hematimetry parameters from baseline to the end of the follow-up period (baseline, 3, 7, 14, 21) in control group (CG) and plasma group (PG). Lines represent the mean ± SEM of the following parameters in each previous mentioned time point: (A) hemoglobin (g/dL), (B) leukocytes (×10^9^/L), (C) neutrophils (×10^9^/L), (D) absolute lymphocytes (×10^9^/L). Figure S2. Changes in hematimetry parameters from baseline to the end of the follow-up period (baseline, 3, 7, 14, 21) in control group (CG) and plasma group (PG). Lines represent the mean ± SEM of the following parameters in each previous mentioned time point: (A) thromboplastin time (seconds), (B) fibrinogen (mg/dL), (C) D-dimer (ng/mL). Figure S3. Changes in hematimetry parameters from baseline to the end of the follow-up period (baseline, 3, 7, 14, 21) in control group (CG) and plasma group (PG). Lines represent the mean ± SEM of the following parameters in each previous mentioned time point: (A) glomerular filtration (mL/min), (B) troponin (ng/mL), (C) procalcitonin (ng/L), (D) C reactive protein (mg/dL), (E) LDH (U/L), (F) IL-6 pg/mL). Figure S4. Correlation between the levels of anti-SARS-CoV-2 S IgG titers (UA/mL) in PG serum vs. donor plasma bag received at: (A) 7, (B) 14, and (C) 21 days after the last infusion. Correlation performed by Pearson test, *p* > 0.05, r^2^ < 0.05. Figure S5. Correlation between the levels of anti-SARS-CoV-2 S IgG titers (UA/mL) in control (CG) and plasma group (PG) serum vs. ACTT score at 21 days after the first infusion (a.i). There were no statistical differences (F(5, 54) = 0.38, *p* > 0.05) by two-way ANOVA followed by the Tukey post hoc test. Table S1. Monitoring procedures and measures carried out during the follow-up period. Table S2. Monitoring procedures and measures carried out during the follow-up period. Table S3. Grades and meanings in the Adaptive COVID-19 Treatment Trial Scale (ACTT-Scale) version II for COVID-19 disease used in the present trial following the Remdesivir Trial and the guidelines of the National Institute of Allergy and Infectious Disease of the United States (NIAID) [15]. Scores considered as clinical improvement are marked in bold. Table S4. Comorbidities in control (CG) and plasma group (PG), with type of diseases and specific diseases, in frequency and percentage.

## Abbreviations

**AAEE**	adverse effect
**BMI**	body mass index
**CG**	control group
**ECMO**	extracorporeal membrane oxygenation
**HP**	hyperimmune plasma
**MOH**	Mateu Orfila Hospital
**PG**	plasma group
**RHM**	Regional Hospital of Manacor
**SEM**	standard error of the mean
**SOFA**	Sequential Organ Failure Assessment score
