# Supplementary material for: Reinforcement of the Standard Therapy with Two Infusions of Convalescent Plasma for Patients with COVID-19: A Randomized Clinical Trial
Source: J Clin Med. 2022 May 27;11(11):3039. doi: 10.3390/jcm11113039 (PMC9181298; doi:10.3390/jcm11113039)
Supplement: Supplementary file 1 [file jcm-11-03039-s001.zip › jcm-1701553-supplementary.pdf]

Supplementary Data

Hematimetry

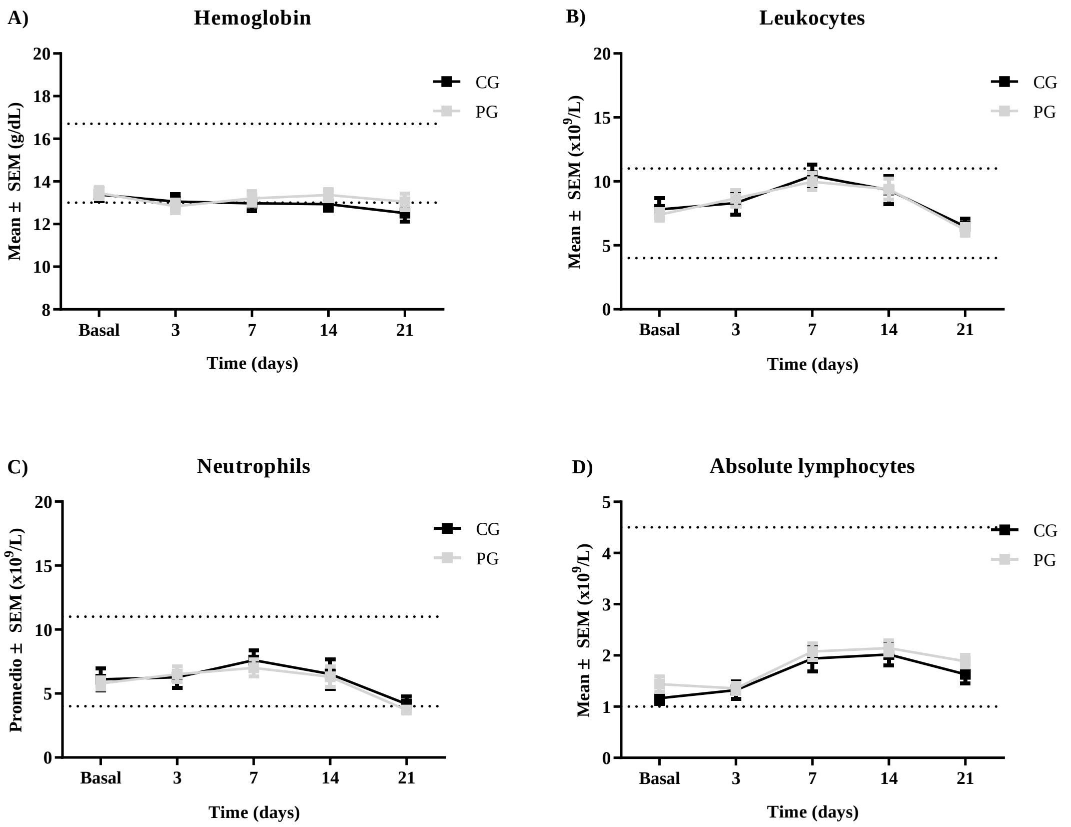

Figure S1. Changes in hematimetry parameters from baseline to the end of the follow-up period (baseline, 3, 7, 14, 21) in control group (CG) and plasma group (PG). Lines represent the mean ± SEM of the following parameters in each previous mentioned time point: (**A**) haemoglobin (g/dL), (**B**) leukocytes (×10⁹/L), (**C**) neutrophils (×10⁹/L), and (**D**) absolute lymphocytes (×10⁹/L).

Coagulation

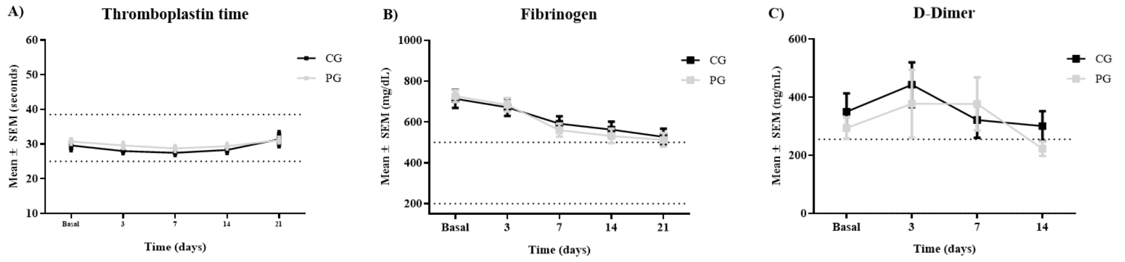

Figure S2. Changes in hematimetry parameters from baseline to the end of the follow-up period (baseline, 3, 7, 14, 21) in control group (CG) and plasma group (PG). Lines represent the mean ± SEM of the following parameters in each previous mentioned time point: (**A**) thromboplastin time (seconds), (**B**) fibrinogen (mg/dL), and (**C**) D-dimer (ng/mL).

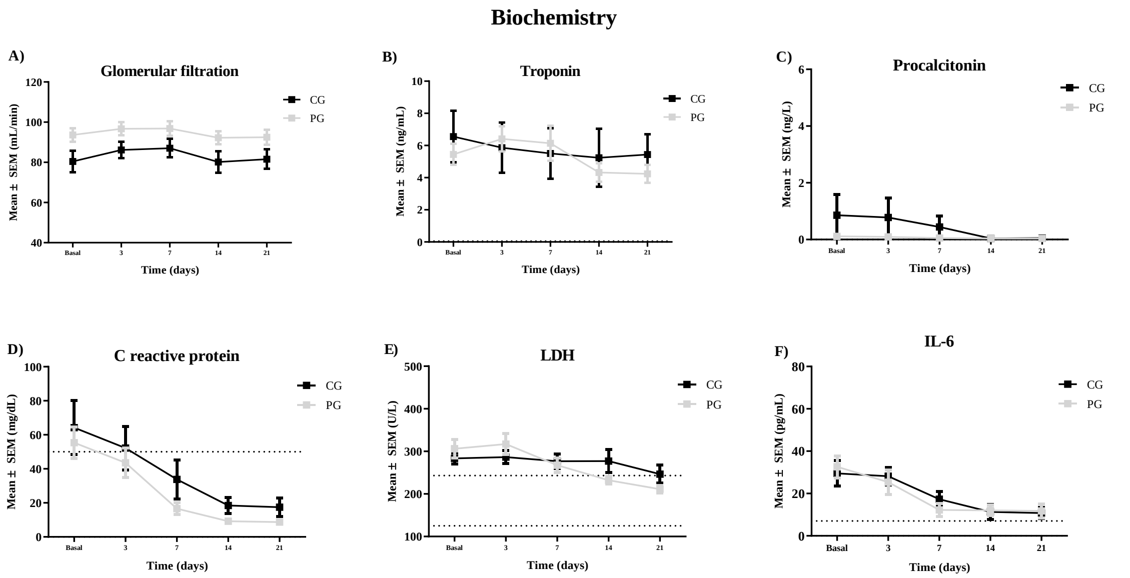

Figure S3. Changes in hematimetry parameters from baseline to the end of the follow-up period (baseline, 3, 7, 14, 21) in control group (CG) and plasma group (PG). Lines represent the mean ± SEM of the following parameters in each previous mentioned time point: (**A**) glomerular filtration (mL/min), (**B**) troponin (ng/mL), (**C**) procalcitonin (ng/L), (**D**) C reactive protein (mg/dL), and (**E**) LDH (U/L), (**F**) IL-6 pg/mL).

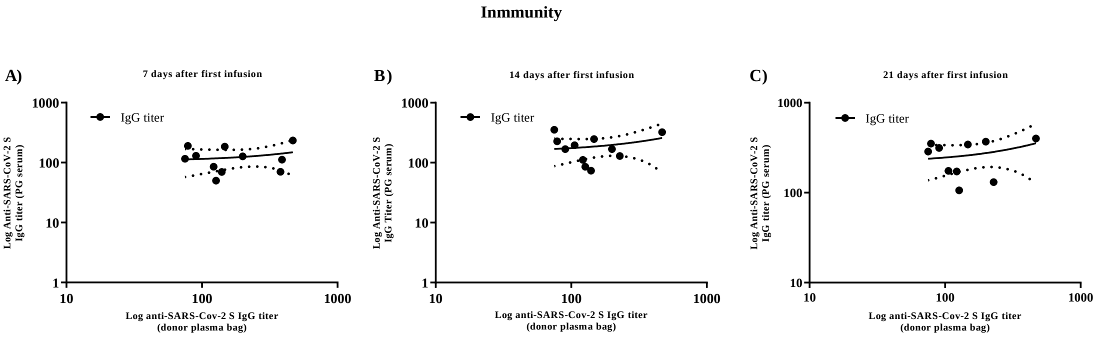

Figure S4. Correlation between the levels of anti-SARS-CoV-2 S IgG titers (UA/mL) in PG serum vs. donor plasma bag received at: (**A**) 7, (**B**) 14, and (**C**) 21 days after the last infusion. Correlation performed by Pearson test, $p > 0.05$, $r^2 < 0.05$.

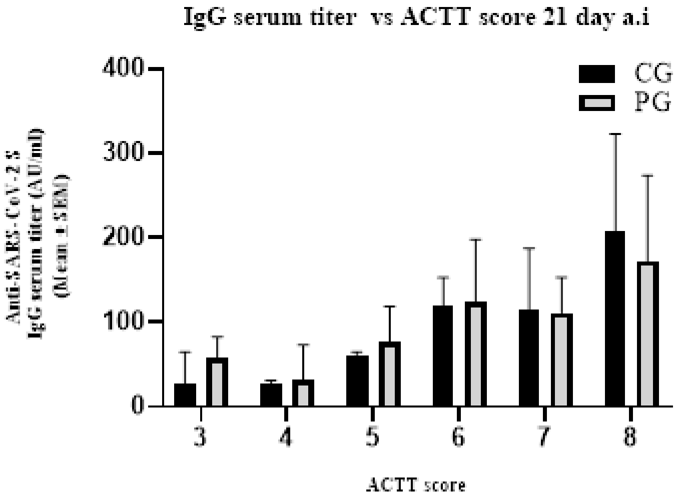

Figure S5. Correlation between the levels of anti-SARS-CoV-2 S IgG titers (UA/mL) in control (CG) and plasma group (PG) serum vs. ACTT score at 21 days after the first infusion (a.i). There were no statistical differences (F(5, 54) = 0.38, $p > 0.05$) by two-way ANOVA followed by the Tukey post hoc test.

Table S1. Standard therapy used in control (CG) $n = 17$ and plasma group (PG) $n = 37$. Type of medication classified by their function, specific drugs administered with distribution in frequency and percentage. χ^2 test was performed to assess statistical differences between drugs and groups.

Type of Medication	Drugs	CG *n* (%)	PG *n* (%)	*p*-Value
Antiaggregant	heparin<br>enoxaparin	5 (29.41)	13 (35.14)	*p* > 0,05
Antibiotics	ceftriaxone<br>azithromycin<br>amoxiciliin-clavulanic acid	5 (29.34)	17 (45.95)	*p* > 0,05
Anti-inflammatory inmunosupressor	desametaxone<br>methylprednisolone	13 (76.47)	32 (86.5)	*p* > 0,05
Antivirals	lopinavir/ritonavir<br>remdesivir	6 (35.29)	12 (32.43)	*p* > 0,05
Oxygentherapy		11 (64.71)	20 (54.05)	*p* > 0,05

Table S2. Monitoring procedures and measures carried out during the follow-up period.

Procedures	Follow-up day						
	**Screening/Basal**	**1**	**2**	**3**	**7**	**14**	**21**
Medical history review and physical exploration	x	x	x	x	x	x	x
Signed informed consent form/randomization	x						
Vital sings	x	x		x	x	x	x
Weight. Height. BMI	x	x		x	x	x	x
ACTT scale	x	x		x	x	x	x
ABO/Rh Group	x						
Hematrimetry	x	x		x	x	x	x
Coagulation	x	x		x	x	x	x
Biochemistry	x	x		x	x	x	x
Serology IgG anti-SARS-CoV-2	x			x	x	x	x
RT-PCR RNA S protein SARS-CoV-2	x			x	x	x	x
Advers effects				x	x	x	x
Concomitant Review and Clinical Response	x			x	x	x	x

Table S3. Grades and meanings in the Adaptive COVID-19 Treatment Trial Scale (ACTT-Scale) version II for COVID-19 disease used in the present trial following the Remdesivir Trial and the guidelines of the National Institute of Allergy and Infectious Disease of the United States (NIAID) [15]. Scores considered as clinical improvement are marked in bold.

Scale	Grade	Meaning
ACTT-version II	1	Death
	2	Hospitalized. on invasive mechanical ventilation or extracorporeal membrane oxygenation (ECMO)
	3	Hospitalized. on non-invasive ventilation or high flow oxygen devices
	4	Hospitalized. requiring supplemental oxygen
	5	Hospitalized. not requiring supplemental oxygen - requiring ongoing medical care (COVID-19 related or otherwise)
	**6**	**Hospitalized. not requiring supplemental oxygen - no longer requires ongoing medical care**
	**7**	**Not hospitalized. limitation on activities and/or requiring home oxygen**
	**8**	**Not hospitalized. no limitations on activities**

Table S4. Comorbidities in control (CG) and plasma group (PG), with type of diseases and specific diseases, in frequency and percentage.

Type of disease	Disease	CG (*n* = 17)		PG (*n* = 37)	
		n	%	n	%
Cardiac diseases	arterial hypertension	11	64.7	26	70.3
	heart disease	4	23.5	2	5.4
	auricular fibrillation	1	5.9	4	10.8
	TOTAL	16	94.1	32	86.5
Respiratory diseases	ASMA	4	23.5	5	13.5
	Sleep apnea	1	5.9	2	5.4
	EPOC	1	5.9	3	8.1
	TOTAL	6	35.3	10	27.0
Digestive diseases	gastritis	2	11.8	2	5.4
	gastroesophageal reflux disease	1	5.9	1	2.7
	TOTAL	3	17.6	3	8.1
Neurological diseases	migraine	2	11.8	7	18.9
	innespecific shaking	1	5.9	0	0.0
	parkinson	1	5.9	0	0.0
	dementia	1	5.9	1	2.7
	TOTAL	5	29.4	8	21.6
Psychiatric diseases	depression	2	11.8	1	2.7
	anxiety	3	17.6	1	2.7
	TOTAL	5	29.4		0.0
Nephrological diseases	prostatic hyperplasia	2	11.8	1	2.7
	renal insuficiency	1	5.9	7	18.9
	TOTAL	3	17.6	8	21.6
Oftalmological diseases	eye infection	1	5.9	1	2.7
	glaucoma	1	5.9	0	0.0
	TOTAL	2	11.8	1	2.7
Dermatological diseases	psoriasis	1	5.9	1	2.7
	TOTAL	1	5.9		0.0
Endocrinological diseases	dyslipidemia	6	35.3	12	32.4
	diabetes miellitus	2	11.8	9	24.3
	hypothyroidism	1	5.9	3	8.1
	obesity	5	29.4	9	24.3
	hypercholesterolemia	1	5.9	3	8.1
	TOTAL	15	88.2	36	97.3
Rheumatic and osteoarticular diseases	cervicoarthrosis	1	5.9	0	0.0
	ankylosing spondylitis	1	5.9	1	2.7
	spondyloarthrosis. dorsal hyperostosis	1	5.9	1	2.7
	osteoarthritis	1	5.9	1	2.7
	arthritis	1	5.9	2	5.4
	TOTAL	5	29.4	5	13.5
Inmunitary diseases	Sjögren syndrome	1	5.9	0	0.0
	TOTAL	1	5.9	0	0.0
